# ROS impairs tumor vasculature normalization through an endocytosis effect of caveolae on extracellular SPARC

**DOI:** 10.1186/s12935-023-03003-8

**Published:** 2023-08-01

**Authors:** Ye Zhao, Jing Yu, Ai Huang, Qin Yang, Guiling Li, Yong Yang, Yeshan Chen

**Affiliations:** 1 Cancer Center, Union Hospital, Tongji Medical College, Huazhong University of Science and Technology, Wuhan, Hubei 430022 China; 2 Institute of Radiation Oncology, Union Hospital, Tongji Medical College, Huazhong University of Science and Technology, Wuhan, 430022 China; 3 Department of Radiation and Medical Oncology, Hubei Key Laboratory of Tumor Biological Behaviors, Hubei Cancer Clinical Study Center, Zhongnan Hospital of Wuhan University, Wuhan, China; 4 Department of Radiation Oncology, Fujian Medical University Union Hospital, Fuzhou, 350001 China

**Keywords:** ROS, Vasculature normalization, Endocytosis effect, Caveolae SPARC

## Abstract

**Background:**

The accumulation of reactive oxygen species (ROS) in tumor microenvironment (TME) is an important player for tumorigenesis and progression. We aimed to explore the outcomes of ROS on tumor vessels and the potential regulated mechanisms.

**Methods:**

Exogenous H_2_O_2_ was adopted to simulate the ROS setting. Immunofluorescence staining and ultrasonography were used to assess the vascular endothelial coverage and perfusions in the tumors inoculated with Lewis lung cancer (LLC) and melanoma (B16F10) cells of C57BL/6 mice, respectively. ELISA and western-blot were used to detect the expression of secreted acidic and cysteine-rich protein (SPARC) and Caveale-1 in human umbilical vein endothelial cells (HUVEC) extra- and intracellularly. Intracellular translocation of SPARC was observed using electron microscopy and immunofluorescence approaches.

**Result:**

Under the context of oxidative stress, the pericyte recruitment of neovascularization in mouse lung cancer and melanoma tissues would be aberrated, which subsequently led to the disruption of the tumor vascular architecture and perfusion dysfunction. In vitro, HUVEC extracellularly SPARC was down-regulated, whereas intracellularly it was up-regulated. By electron microscopy and immunofluorescence staining, we observed that SPARC might undergo transmembrane transport via caveale-1-mediated endocytosis. Finally, the binding of SPARC to phosphorylated-caveale-1 was also detected in B16F10 tissues.

**Conclusion:**

In the oxidative stress environment, neovascularization within the tumor occurs structural deterioration and decreased perfusion capacity. One of the main regulatory mechanisms is the migration of extracellular SPARC from the endothelium to intracellular compartments via Caveolin-1 carriers.

## Background

Carcinogenesis requires a complex microenvironment. Unlike the normal context, the tumor microenvironment (TME) is often accompanied by significant adverse elements that can easily trigger tumor cells to resist drugs or irradiation and subsequently elicit recurrence or metastasis [1–5]. Blocking detrimental components in TME, therefore, may mitigate their therapeutic resistance.

Aberrant angiogenesis is an important feature of the TME. It has been demonstrated in multiple tumor models that tumor parenchymal cells, as well as the mesenchyme components overexpress pro-angiogenic factors that contribute to the formation of aberrant vascular nets characterized by disorganization, immaturity and permeability that underpin the hypoxia and poor perfusion in TME [6, 7]. Hypoxia in turn exacerbates vascular aberrations while promoting the aggressiveness of tumor cells and counteracting the killing of lymphocytes, such as CD8+ T cells, etc. [8–10]. In addition, the distorted tumor vessels often impede drug delivery and compromise the response to irradiation [11–13]. Therefore, vasculature normalization is an important strategy to reduce the aggressiveness of tumor cells, as well as to re-sensitize them to treatments.

In the context of TME, reactive oxygen species (ROS) are cumulatively elevated in response to the combined stimulus of a hypoxic background and some other adverse stimuli, which are detrimental to treatment [14, 15]. In previous studies, we found that high doses of lipid-lowering drug simvastatin can improve ROS-dependent tumor vascular hyperpermeability in mouse models of lung cancer and melanoma [16]. In this study, we would further explore the specific mechanisms of ROS scavenging on tumor vasculature normalization and its potential targets.

## Materials and methods

### Cell line and cell culture

Lewis lung cancer (LLC), melanoma (B16F10) cells and Human umbilical vein endothelial cells (HUVEC) were purchased from American Type Culture Collection (ATCC; Rockville, MD, USA). B16F10 and LLC cells were cultured in RPMI-1640 (HyClone, US) supplemented with 10% FBS (HyClone, US) and 100U/ml penicillin and 0.1 mg/ml streptomycin (HyClone, US). HUVECs were sustained in endothelial cell medium (ECM; ScienCell, CA) adding EC growth supplements and 5% FBS. All cells were incubated in a humidified atmosphere of 5% CO_2_ at 37 °C.

### Animal tumor model

LLC (2 × 10^6^) and B16F10 cells (2 × 10^5^) were injected s.c. into the right flank of 8-weeks-old female C57BL/6 mice (Animal Care Committee of Wuhan university, Wuhan, China). Treatments were initiated when tumors reached a size of ~ 100 mm^3^. Animals were randomly selected to receive H_2_O_2_ (50nmol/kg/d, Sigma, US), H_2_O_2_ (12.5nmol/kg/d), 0.9% NaCl solution and DPI (1 mg/kg/d, Sigma, USA) for 5 days. Tumor-bearing mice were euthanized using 2% pentobarbital sodium and all animal procedures were approved by the Animal Care Committee of Wuhan Union Hospital, Huazhong University of Science and Technology (SYXK 2010-0057).

### Immunofluorescence

Subcutaneously implanted LLC and B16F10 tumor tissues were harvested and immediately frozen with optimal cutting temperature (OCT) compounds (Sakura, Japan). Tumor sections (40 μm thick) were co-immunostained with goat anti-CD31 (1:50; Biotechnology, China) and mouse anti-α-smooth muscle actin (α-SMA) (1:200; Google Biotechnology, China) antibodies, followed by Cy3-conjugated donkey anti-goat secondary antibody (1:300; Google Biotechnology, China) as well as FITC-conjugated donkey anti-mouse secondary antibody (1:200; Google Biotechnology, China). Images were captured using a confocal microscope (Zeiss, Germany). The pericyte coverage index was calculated as the percentage of vessels positive for CD31 and α-SMA staining. All images were analyzed with Image-Pro Plus 6.0 software (Media Cybernetics, MD).

### Fluorescein perfusion test

Perfusion of tumoral vessels was assessed by tail vein injection of 0.05 mg FITC-labeled lectin (1 mg/ml in 0.9%NaCl, Sigma, USA). After completion of fluorophores injection, it was necessary to allow 10 min of circling in mice, and then the tumors were harvested and immediately frozen in a dark environment by OCT compound. Tumor sections (40 μm) were prepared and incubated with goat anti-CD31 antibody (1:50) overnight at 4 °C, followed by incubation with Cy3-conjugated secondary antibody (1:200). The perfusion function of tumor vessels was determined by calculating the percentage of FITC-lectin+/CD31+ stained vessels.

### Ultrasonography

The vascular perfusion signal within the tumor was assessed using a color Doppler flow imaging system of a 1–5 MHz probe (iU22 SonoCT, Philips, Netherlands). Tumor-bearing mice were anesthetized and placed on an electric heating plate at 37 °C. For selecting the region of interest (ROI), the blood flow signal was taken on the corresponding tumor sections and analyzed by QLAB quantitative technique. The amount and intensity of tumor vascular perfusion signals were assessed by vascularization index (VI) and vascularization flow index (VFI), respectively.

### Cell counting Kit-8 assay

HUVECs were seeded at 8000 cells/well in 96-well plates and grown overnight with attached walls. The cells were treated with H_2_O_2_ at concentrations of 0, 50µM, 100µM, 250µM, 500µM, 1000µM, 2500µM, and 5000µM for 30 min, 6 h, and 24 h, respectively. Cholecystokinin-8 (Shangbo Biotechnology, China) was added to each well (10 ul per well) and incubated for 2 h at 37 °C. The optical density (OD) was measured at 450 nm by using a scanning multi-well spectrophotometer (Bio-Rad Model 550, CA, United States).

The cell inhibition rate was calculated using the following formula: cell inhibition rate = [(OD control - OD experimental group) / OD control group] × 100%. Cells were treated with different concentrations of H_2_O_2_ and assayed at three time points, with each repeated thrice.

### Enzyme-linked immunosorbent assay (ELISA)

Human SPARC ELISA kits (Shenglong Biotechnology, China) were used to measure the amount of SPARC and matrix metalloproteinase 2 (MMP-2) in the medium of HUVECs. The experimental procedure was performed according to the instructions. The optical density (OD) was assayed at 450 nm using a scanning multi-well spectrophotometer (Bio-Rad Model 550, CA, US).

### Gelatin zymography

HUVECs were seeded at 1.0 × 10^6^ cells/well in 6-well plates and allowed to grow attached to the wall overnight. Cells were then incubated continuously with serum-free medium for 12 h, after which HUVECs were treated with 0 and 250µM of H_2_O_2_ for 24 h. Finally, the medium was collected and centrifuged. The BCA method was used to determine the protein concentration in the culture medium, which was used to adjust the amount of protein samples. APMA, 4-Aminophenylmercuric acetate, was selected as a positive control. The gel was subjected to two shaking elution for 20 min each, followed by two rinses of 20 min each with the rinse solution (Triton X-100 free in the eluent). Then it was put in the incubation solution on the shaker for 42 h. After 3 h of staining, the gels were dyed blue and then treated with 10% decolorizing solution for 0.5 and 1 h and 5% decolorizing solution for 2 h, respectively. Ultimately, the locations where the white and translucent bands appear were observed. All images were analyzed by Quantity One software (Media Cybernetics, MD).

### Western-blot analysis

To detect the expression levels of SPARC, pY14-caveolin-1 and PDGF-BB in tumor tissues, equal quantities of protein extracts were loaded onto 12% SDS-PAGE and transferred to nitrocellulose membranes (Millipore, Billerica, MA). After blocking with 5% casein/TBST, the membranes were incubated with rabbit anti-SPARC (1:100; CST, US), rabbit anti-pY14-caveolin-1 (1:50), and rabbit anti-PDGF-BB (1:200; Abcam, US). The mouse anti-β-actin (1:1,000; Google Biotechnology, China) antibody was used as the internal control. Protein blots were visualized using Super Signal Chemiluminescent kit (Google Biotechnology, China). The chemiluminescent signals on X-ray film were scanned and analyzed by AlphaEaseFC software (Innotech, US).

### Electron microscope

Prior to electron microscopy assay, HUVECs were seeded at 1 × 10^6^ cells/well in 6-well plates and grown overnight with adherent walls. Afterwards, the cell samples were treated with 0 and 250µM H_2_O_2_ for 30 min, respectively. Samples were collected and fixed with electron microscope stationary liquid, and stored at 4 °C for 3 days. Samples were washed three times with 0.1 M PBS, then soaked in a solution containing osmium tetroxide (1%) for 1 h, and then washed again with PBS. After a further staining in 1% aqueous uranyl acetate for 30 min, those samples were dehydrated and embedded in 50%, 70%, 90%, 100% ethanol and anhydrous acetone for 20 min, respectively. And then the cells were treated with a 1:1 volume mixture of anhydrous acetone and embedding agent and shaken for 2 h. Thereafter, the cells were treated with pure embedding agents and shaken for 2 h. The embedding agents were polymerized in an oven as the following conditions: 37 °C for 24 h, 45 °C for 24 h, and 60 °C for 48 h. A thin slice (120 nm) was made and the caveolae structure on the surface of HUVECs were observed by using transmission electron microscopy (80 kV, 15,000×; JEOL, Japan).

### Co-localization

HUVECs sections were co-immunostained with rabbit anti-PY14-caveolin-1 (1:100) and goat anti-SPARC (1:100) antibodies, and then treated with Cy3-conjugated donkey anti-rabbit secondary antibody (1:100) and FITC-conjugated donkey anti-goat secondary antibody (1:200). The co-immunostaining index of pY14-caveolin-1 and SPARC was estimated using Image-Pro Plus 6.0 software (Media Cybernetics, MD).

### FITC marked hr-SPARC

We use HOOK™ Dye Labeling Kit (FITC) (Sagan, China) to mark Human recombinant SPARC proteins (RnD, USA). Prior to labeling treatment, 100 µl of DMSO was added to the dye marker and mixed well. Then, the dye marker was mixed thoroughly with the protein solution and centrifuged, and the samples were collected from the bottom of the tube. Thereafter, those samples were wrapped in aluminum foil and incubated at room temperature for 60 min. Subsequently, we collected and purified the protein samples according to the detailed process instructions of the HOOK™ Dye Labeling Kit (FITC) and stored them for backup.

### Internalized inhibition

HUVECs were seeded in 6-well plates at 1 × 10^6^ cells/well and allowed to grow in adherent walls overnight, the culture medium was replaced with serum-free medium, followed by addition of 5 µg/ml FITC-SPARC. We then treated HUVECs with 250µM H_2_O_2_ and 5 µg/ml Filipin-III (Caveolae/caveolin-1 internalization pathway inhibitor; Cayman, China) for 1 h, respectively.

### Statistical analysis

All measurements were repeated at least three times. Quantitative data were presented as mean ± standard deviation (SD). Significant differences between two groups were analyzed by Student’s t test (normally distributed data) or Mann-Whitney U test (non-normally distributed data). For comparisons across three or more treated groups, the analysis of variance (ANOVA) and the Wilcoxon rank sum test were applied for normal distribution data and non-normal distribution data, respectively. All calculations were performed using SPSS Statistics software, version 22.0 (IBM Corporation, Armonk, NY). Statistical significance was set at a two-sided *P* < 0.05.

## Results

### ROS disrupts the vessel structure

Vascular endothelial cells and mature pericytes are important components of normal blood vessels; therefore, we first determined the status in vascular structures by detecting the expression of CD31, a marker of endothelial cells, and α-SMA, the marker of pericytes, at different ROS levels in LLC and B16F10 mouse models. We gave mice intraperitoneal H_2_O_2_ injections at different concentrations to simulate different intensities of the oxidative stress environment.

When the grafts grew uniformly to 100 mm^3^ (6 days for B16 and 8 days for LLC), the two types of mice were randomly grouped into control, low ROS (H_2_O_2_, 12.5 nmol/kg, once daily) and high ROS level (H_2_O_2_, 50 nmol/kg, once daily) groups, and DPI (diphenyleneiodonium) de-oxidation treated group (1 mg/kg, once daily). After 5 days of interventions, we collected the transplanted tumors for further detection. No death as well as an abnormal weight loss of the mice was observed during the course of intervention.

**Fig. 1:**
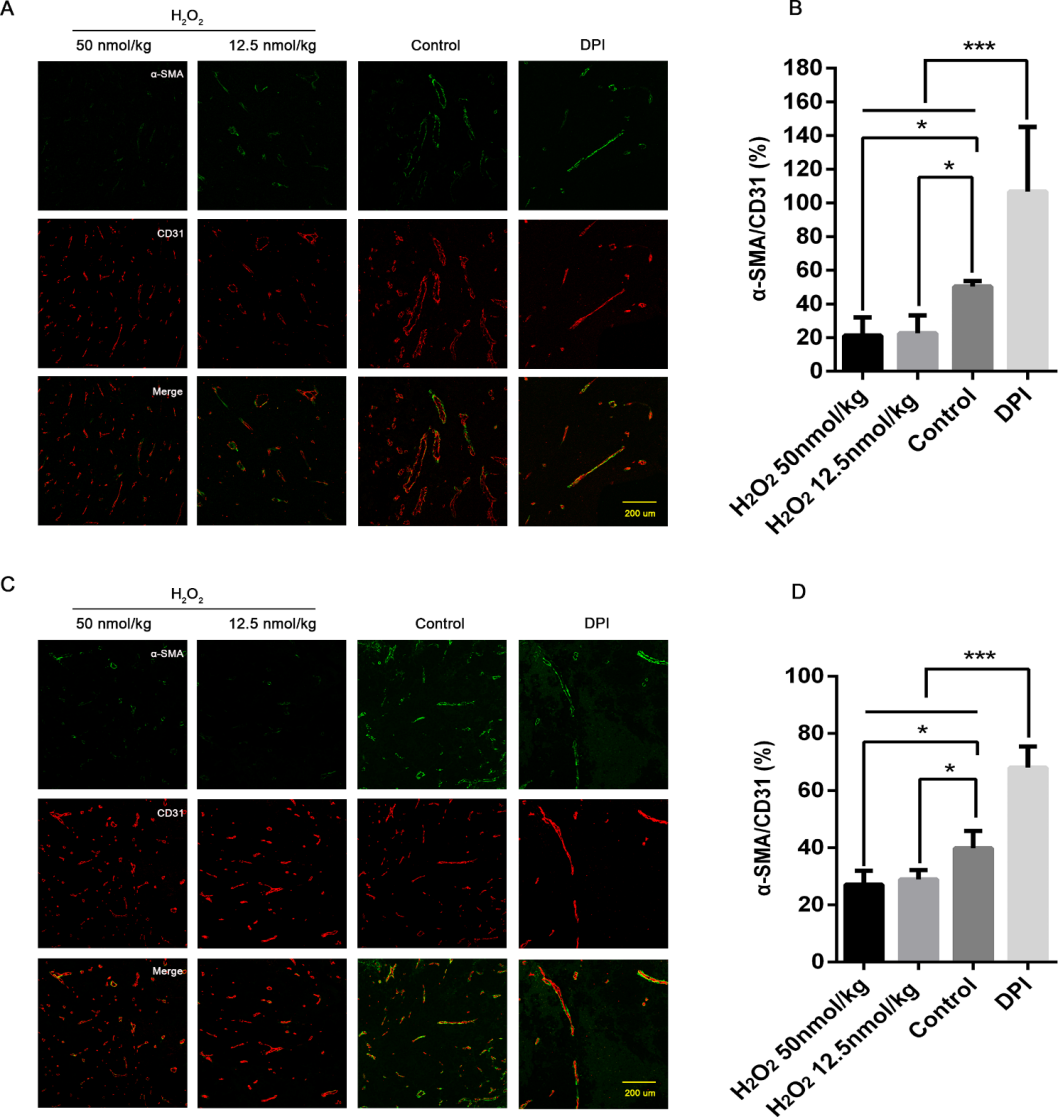
In comparison with the control group, the density of vessels with normal structure was significantly reduced in LLC (A&B) and B16F10 (C&D) tumor tissues after H_2_O_2_ treatments (α-SMA+ vascular smooth muscle cell, green; CD31+ endothelial cells, red). Data presented were mean ± SD. By DPI treatment, a higher proportion of normal structured blood vessels could be observed. Differences among two H_2_O_2_-treated and DPI-treated groups were tested by ANOVA, with the * representing p values less than 0.05 and the *** representing p values less than 0.01 LLC: Lewis lung carcinoma cell line; B16F10: murine melanoma cell line; DPI: diphenyleneiodonium; ANOVA: analysis of variance

Compared to controls, a low proportion of α-SMA+/CD31+ vessels could be detected in the LLC xenograft model with either low or high H_2_O_2_ treatments. A significant recovery of those vessels occurred by DPI antioxidant treatments (Fig. 1A-B). Likewise, under the same conditions of H_2_O_2_ as well as a recovery method, a similar change of α-SMA+/CD31+ vessels could be observed in the model B16F10 (Fig. 1C-D).

### ROS impairs vascular perfusion function

**Fig. 2:**
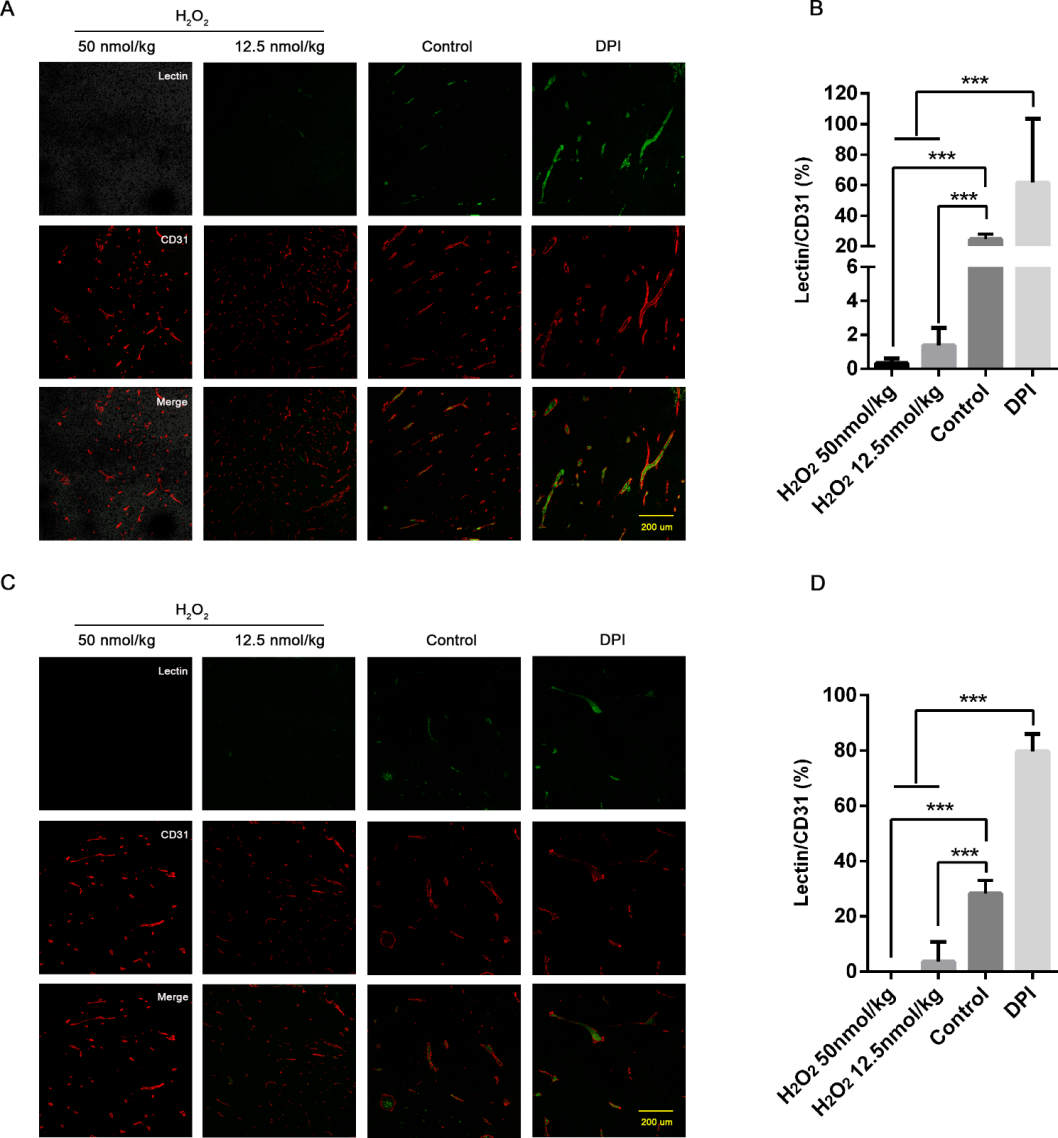
Perfusion function of vessels within LLC (A&B) and B16F10 (C&D) tissues was significantly impaired after H_2_O_2_ treatment when compared to the controls (FITC-lectin+, green; CD31+ endothelial cells, red). Data presented were mean ± SD. After treating with DPI, the proportion of vessels with normal perfusion function was significantly increased (*P* < 0.01). Differences among two H_2_O_2_-treated and DPI-treated groups were tested by ANOVA, with the *** representing p values less than 0.01 LLC: Lewis lung carcinoma cell line; B16F10: murine melanoma cell line; DPI: diphenyleneiodonium; ANOVA: analysis of variance

We took FITC-lectin tail vein injection to observe the perfusion function of vessels in both models under the oxidative stress background. In response to H_2_O_2_ (both low or high concentration backgrounds), the density of normally perfused vessels (indicating Lectin+/CD31+ staining) in LLC was markedly reduced as compared to controls, and those could be significantly elevated with DPI antioxidant treatment (Fig. 2A-B). We then adopted the same strategy for the validation in the B16F10 model, and observed a consistent change (Fig. 2C-D).

**Fig. 3:**
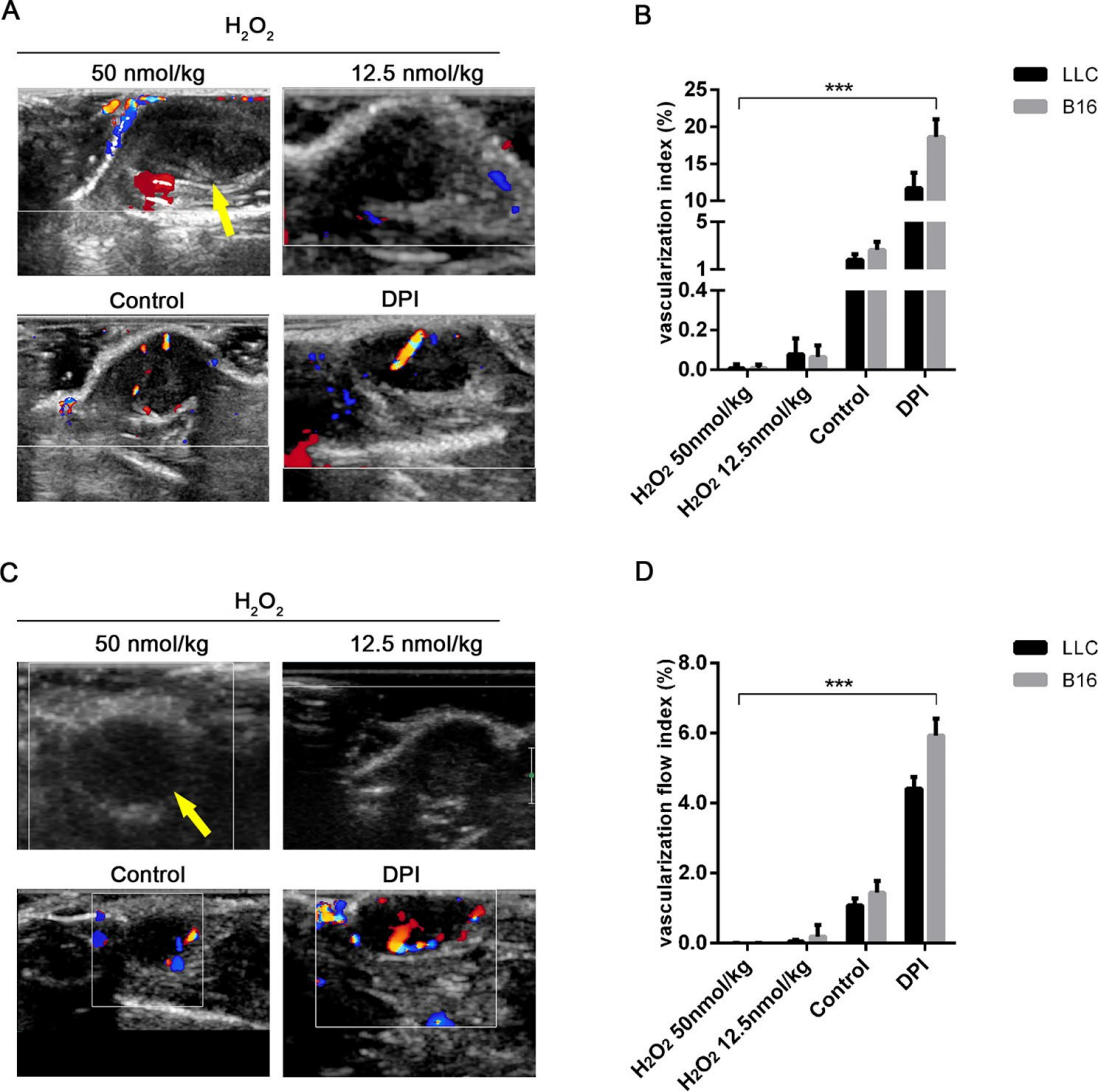
Ultrasonography showed that VI and VFI within tumor tissues were significantly decreased after the H_2_O_2_ treatments when compared to the control group. After treating with DPI, the perfusion signals were significantly upregulated. The yellow arrow indicated the tumor areas; A&B figure was LLC model while C&D figure represented B16F10 model. Data presented were mean ± SD. Differences of VI and VFI among treatment groups were assessed by ANOVA, *** represents p- value less than 0.01 VI: vascularization index; VFI: vascularization flow index; LLC: Lewis lung carcinoma cell line; B16F10: murine melanoma cell line; ANOVA: analysis of variance

Furthermore, a color Doppler ultrasound method was performed to visually investigate the perfusion of tumoral vessels. As seen from Fig. 3A and C, for both LLC and B16F10, few blood flow signals could be detected within tumors relative to controls, in response to H_2_O_2_. After DPI treated, high levels of flow signal could be measured.

The strength of the vascular signal could be quantified by VFI and VI. Accordingly, H_2_O_2_ decreased both VFI and VI in those two models, which, significantly, were improved by DPI antioxidant treatment, highlighting the negative role of oxidative stress on the vascular perfusions (Fig. 3B-D).

Combined with the results above, it is reasonable to assume that the impairment of vascular perfusion in conditions of H_2_O_2_ is mainly attributed to the abnormal recruitment of pericytes.

### The role of SPARC under ROS conditions

**Fig. 4:**
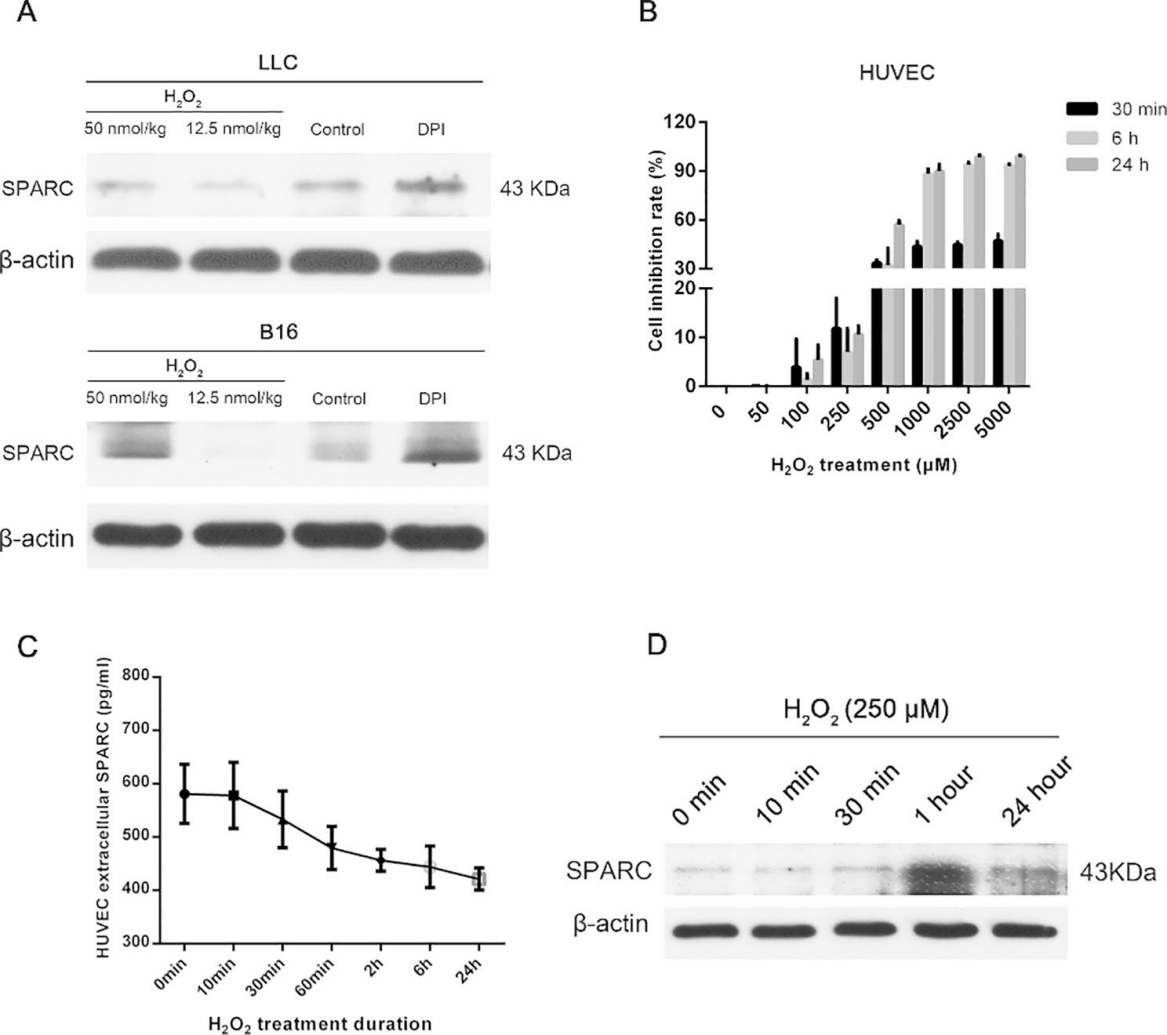
Western-blot showed the protein expression of SPARC was reduced in LLC and B16F10 tissues treated with H_2_O_2_ as compared to controls, while DPI treatment resulted in an up-regulation of SPARC (A); Cell inhibition rate of HUVECs at different treatment time points (30 min, 6 and 24 h) across a range of H_2_O_2_ intensities (B); ELISA demonstrated that SPARC of HUVECs decreased with the duration of H_2_O_2_ exposure (C); Western-blot showed a significant upregulation of intracellular SPARC in the presence of H_2_O_2_. SPARC: secreted acidic and cysteine-rich protein; LLC: Lewis lung carcinoma cell line; B16F10: murine melanoma cell line; HUVEC: human umbilical vein endothelial cells; ELISA: enzyme-linked immunosorbent assay

As SPARC is thought to be an important regulator of pericyte recruitment [17, 18], we first attempted to investigate the potential role of SPARC in the context of ROS. Western-blot assay from LLC and BF16 tumors showed that SPARC protein would downregulate in the H_2_O_2_ environment when compared to the control group. After removal of ROS by DPI, the level of SPARC could be restored (Fig. 4A).

### ROS decreases extracellular SPARC protein content in HUVEC

HUVECs are one of the most commonly applied models in vitro to characterize vascular biology. Through concentration-dependent experiments, we found that 250 µM H_2_O_2_ inhibited cells more stably at different time points and did not cause excessive cell death, hence determining it as the operating concentration, for in vitro experiments (Fig. 4B).

By ELISA, we observed a linear downregulation of extracellular SPARC with the duration of H_2_O_2_ intervention (Fig. 4C). However, the expression level of SPARC within HUVECs were significantly upregulated along with the treatment of H_2_O_2_ (Fig. 4D). The above results implied that the SPARC protein might have experienced a process of extracellular to intracellular translocation.

### Mechanism of SPARC downregulation in the extracellular matrix of HUVEC under the ROS environment: a caveolae dependent endocytosis

**Fig. 5:**
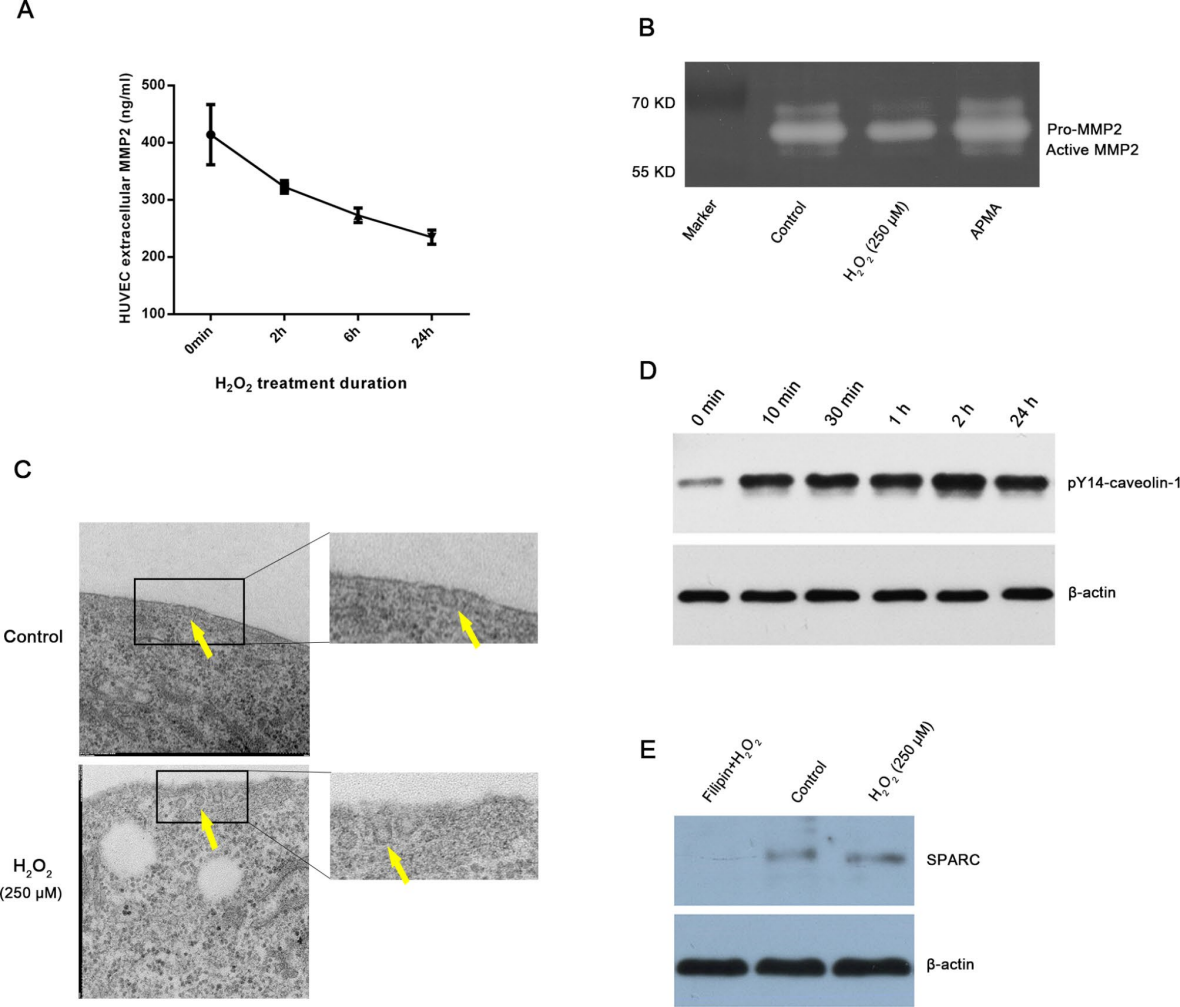
ELISA showed that extracellular MMP-2 of HUVEC decreases with prolonged exposure to H_2_O_2_ (A); Gelatin zymography indicated that H_2_O_2_ caused diminished activity of MMP-2 (B); Electron microscopy observed that the caveolae-1 on the surface of HUVEC were more accessible to the intracellular compartment under the treatment of H_2_O_2_, the yellow arrows indicated the caveolae-1 (C). Western-blot showed that the expression of phosphorylated caveolin-1 within HUVEC was upregulated in response to H_2_O_2_ (D). The addition of filipin-III resulted in downregulation of SPARC protein within HUVECs compared to H_2_O_2_-treated group and control (E) HUVECs: human umbilical vein endothelial cells; ELISA: enzyme-linked immunosorbent assay; MMP-2: matrix metalloproteinase 2; SPARC: secreted acidic and cysteine-rich protein

It has been suggested that extracellular SPARC could be degraded by MMP-2 [19]; therefore, we assayed the quantification and activity of extracellular MMP-2 by ELISA and gelatin zymography assay (Fig. 5A**&B**), both of which were decreased upon H_2_O_2_ treatment, presuming that the extracellular downregulation of SPARC is not driven by the MMP-2 pathway.

Caveolae are bottle-shaped invaginated molecules presented in the plasma membrane of many types of cell, and they are capable of participating in the transport of many molecules across the membrane [20, 21].

Through membrane electron microscopy, we could observe the flask-shaped caveolae were precisely located on the surface of the cell membrane with its opening oriented towards extracellular matrix in the control group. Under the stimulation of H_2_O_2_, increasing number of caveolae internalized gradually and formed intact vesicle-like structures moving toward intracellular matrix (Fig. 5C). Moreover, Western-blot of HUVEC cells also showed that the corresponding phosphorylated Caveolin-1 protein levels were significantly upregulated under the H_2_O_2_ treatment (starting after 10 min and up to 24 h) (Fig. 5D). When adding Filipin-III to HUVECs, an inhibitor of caveolae internalization, it led to a significant reduction of intracellular SPARC (Fig. 5E).

**Fig. 6:**
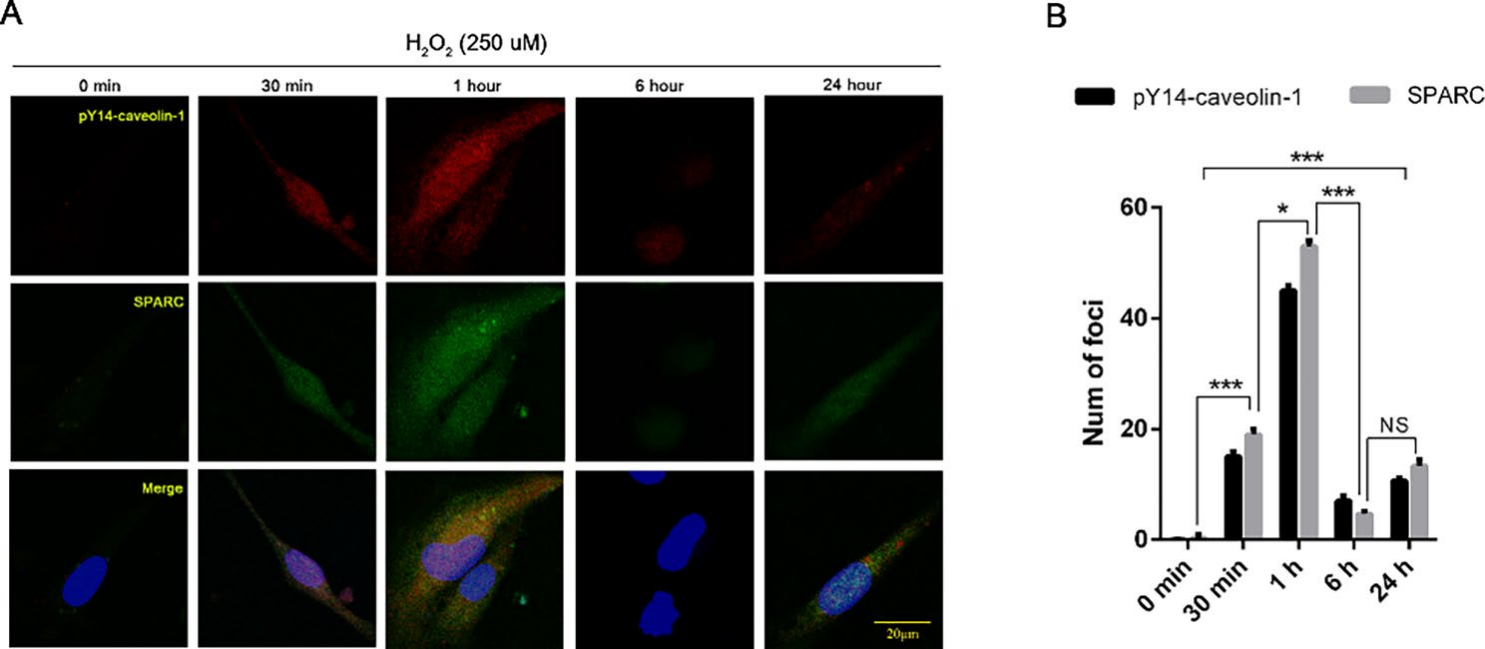
Intracellular co-localized expression of phosphorylated Caveolin-1 (red) and SPARC (green) was significantly increased in HUVECs in response to H_2_O_2_ treatment relative to the control group. Data presented were mean ± SD. Phosphorylated caveolin-1 and SPARC at different H_2_O_2_ treatment time points were compared by ANOVA, * represents p-value less than 0.05, *** represents p-value less than 0.01 HUVECs: human umbilical vein endothelial cells; SPARC: secreted acidic and cysteine-rich protein; ANOVA: analysis of variance

By immunofluorescence co-localization of phosphorylated caveolin-1 and SPARC within HUVEC, we observed that SPARC endocytosed by pY14-caveolin-1 could be detected intracellularly after H_2_O_2_ treatment, further confirming the internalization effect of SPARC (Fig. 6A**&B)**.

### Internalization effect of phosphorylation-activated Caveolin-1 on SPARC proteins in tumor tissues under ROS environment

**Fig. 7:**
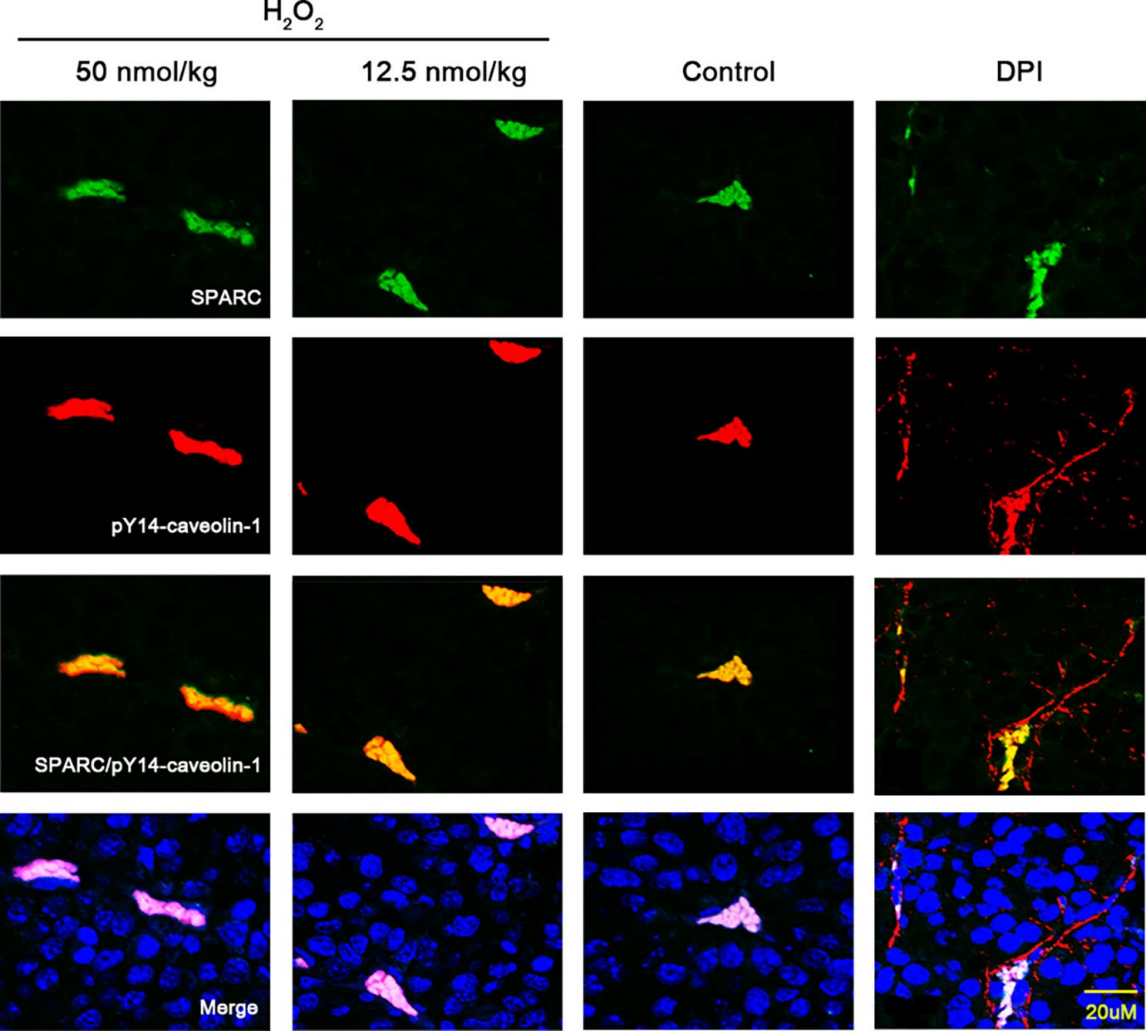
Immunofluorescence co-localized expression of phosphorylated Caveolin-1 (red) and SPARC (green) in the B16F10 tissue under the treatment of H_2_O_2_. B16F10: murine melanoma cell lines

We chose the B16F10 mouse models to verify the endocytosis of phosphorylated caveolin-1 on SPARC in vivo. We found that the expression of pY14-caveolin-1 and SPARC protein was highly overlapped in tumor tissues treated with H_2_O_2_. However, the co-localization of pY14-caveolin-1 with SPARC was significantly weakened when ROS was scavenged. (Fig. 7).

## Discussion

In TME, ROS is capable of initiating multiple processes in tumorigenesis, including the regulation of angiogenesis [22], metastasis [23] and cell apoptosis [24], etc. In our study, using LLC and B16 models, we found that vascular structures and perfusion function within the tumors were compromised under H_2_O_2_ treatments. Through in vitro experiments, we further observed that the detrimental outcomes of ROS on tumor vascular normalization could be mediated through an endocytosis of the caveolin-1 on extracellular SPARC.

Abnormal neovascularization in TME plays an important role in tumorigenesis. First, tumor cells need enough blood to survive, and second, the massive proliferation of immature vessels provides the environment for invasion and metastasis. With an in-depth understanding of the angiogenic blockade, the anti-angiogenic mechanism consists less of simply inhibiting the growth of tumor vessels, but more of promoting the normalization of abnormal structures [25]. Preclinical studies had shown that targeting blockade of VEGF and its receptors, could facilitate normalization of tumor vasculature and remodeling of TME [26, 27]. Clinical studies confirmed that multiple blockers of VEGF in combination with conventional chemotherapy or in triple therapy with immune checkpoint inhibitors would produce synergistic effects and provide survival benefits for multiple tumor types [28, 29].

In oxidative stress conditions, excess ROS are produced, damaging cellular proteins, lipids and DNA, leading to carcinogenesis. For example, H_2_O_2_ could oxidize prolyl hydroxylase domain protein 2 (PHD2) and lead to the stabilization of hypoxia-inducible factor 1α (HIF-1α), a protein that is essential for angiogenesis and cancer metastasis [30]. ROS can also promote the pancreatic cancer formation by activating NF-κB and upregulating EGFR proliferative signaling via protein kinase D1 [31]. In addition, ROS can also cause DNA damage, including single strand cleavage, point mutations, miscoding, abnormal amplification and activation of oncogenes [32]. In this study, we simulated the landscape of oxidative stress with exogenous H_2_O_2_ in both ex vivo and in vitro, and we found that under conditions of overexpressed ROS, the recruitment of pericytes to the vascular endothelium would be compromised, leading to an abnormal vascular architecture. Also, we observed an underlying H_2_O_2_ concentration-dependent decrease of perfusion, and thereby we postulated that vascular structural abnormalities caused by impairment of pericyte were one of the main contributors.

A growing body of evidence suggests that the oxidative stress process is closely associated with Caveolin-1 [33]. Reactive species produced under the oxidative stress could induce the Caveolin-1 expression and facilitate cell membrane internalization of target proteins, for example, In an ischemia-reperfusion model, oxidative stress can induce internalization of P-glycoprotein through phosphorylation of Caveolin-1, reducing its mediated cortisol efflux to the blood-brain barrier to mitigate neuronal injuries [34], Caveolin-1 also increases HDL biogenesis through internalization and degradation of ATP binding cassette transporter (ABCA1), which would be modulated by propanol oxidation products [35]. Caveolin-1 also exerts feedback regulation on the oxidative stress reactions in TME [36].

The pericytes are involved in angiogenesis and are essential for the development of a functional vascular network [37]. They are able to mobilize and migrate from established vessels to new ones through factors such as matrix metalloproteinase 9 (MMP-9) and platelet-derived growth factor B (PDGF-BB). In TME, once pericytes are poorly mobilized, de novo vessels exhibit highly leaky and tortuous features. Targeted improvement of pericyte recruitment would improve the responsiveness to therapy.

SPARC is an extracellular calcium-binding glycoprotein that participates in a variety of cellular processes, including promoting inflammatory cell aging [38], regulating stromal cell differentiation [39], and increasing tumor cell invasion [40]. SPARC is also engaged in pericyte recruitment and neo-angiogenesis [17]. Herein, we observed that under oxidative stress conditions, extracellular SPARC was translocated into cells by membrane-bound protein caveolae-1. Furthermore, in the B16F10 tissue model, we also witnessed that endocytosis, suggesting that internalization of SPARC is a key mechanism of vascular dysfunction.

Next, we will investigate the structure and function of vessels under conditions of blocking or upregulating caveolin-1, and the corresponding SPARC expression, which would shed light on the role of the caveolin-SPARC pathway in tumor angiogenesis scenario.

## Conclusion

**Fig. 8:**
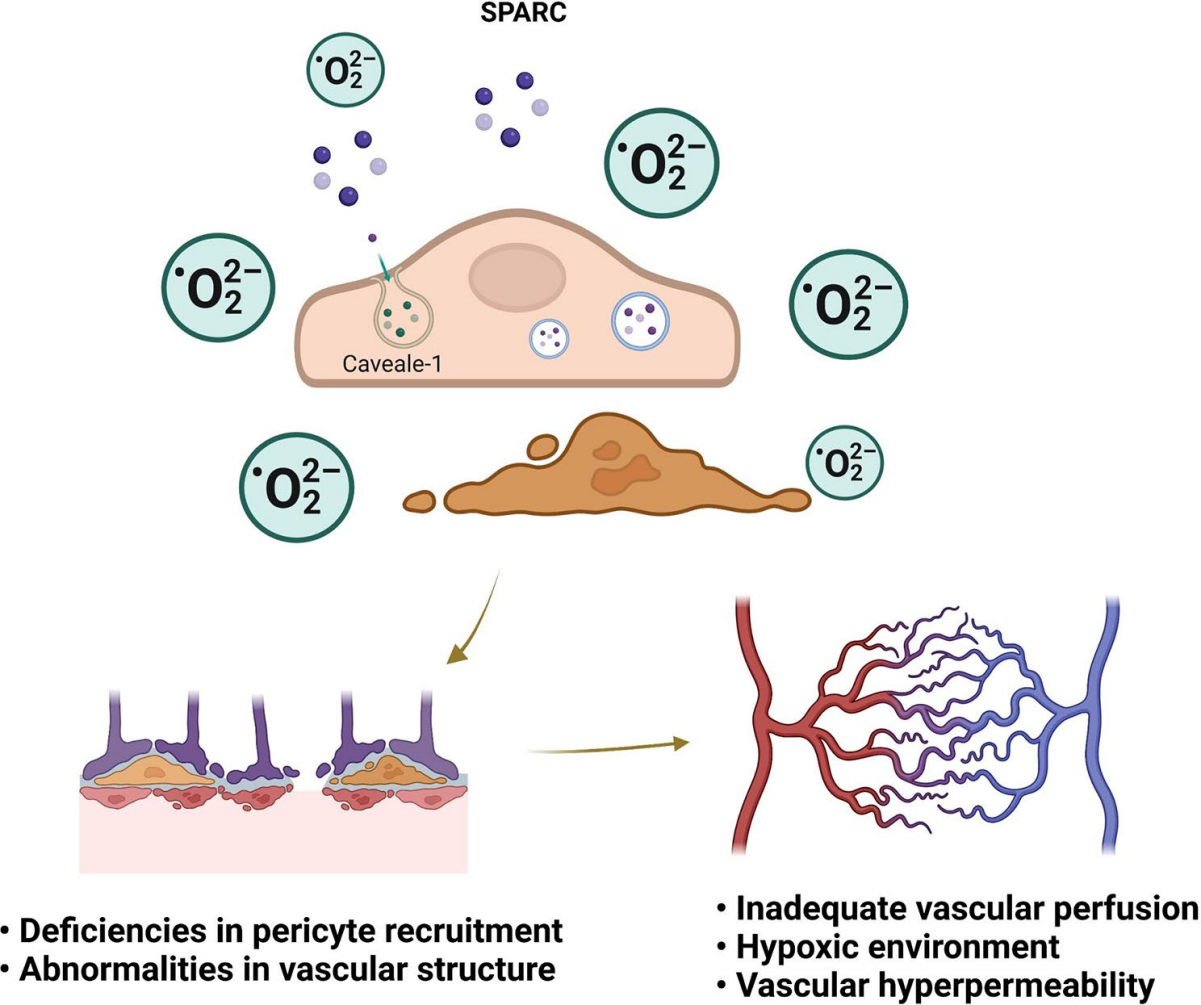
In response to oxidative stress, SPARC outside the endothelium enters intracellularly via endocytosis of caveolin-1, triggering defective recruitment of pericytes, structural aberrations followed by inadequate perfusion, hypoxic microenvironment, and hyperpermeability (Created with BioRender.com). SPARC: secreted acidic and cysteine-rich protein

In TME, high levels of oxidative stress increase endocytosis of SPARC by caveolin-1, leading to inadequate recruitment of pericytes and causing distortion of intertumoral vascular function (Fig. 8). Amending the high oxidative stress in TME or targeting caveolin-1 may induce vascular normalization and improve efficacy.

## Data Availability

The data used to support the results of this study are available through the Institutional Review Board and the authors
